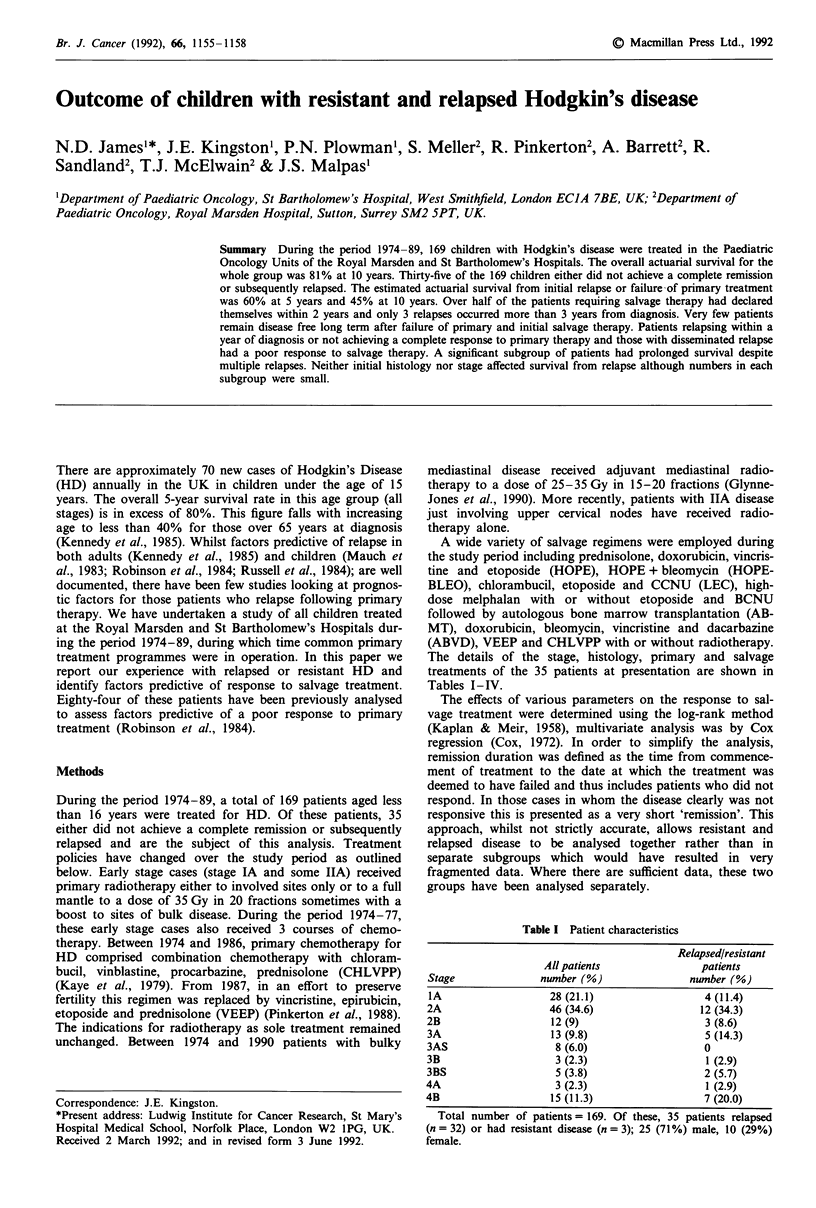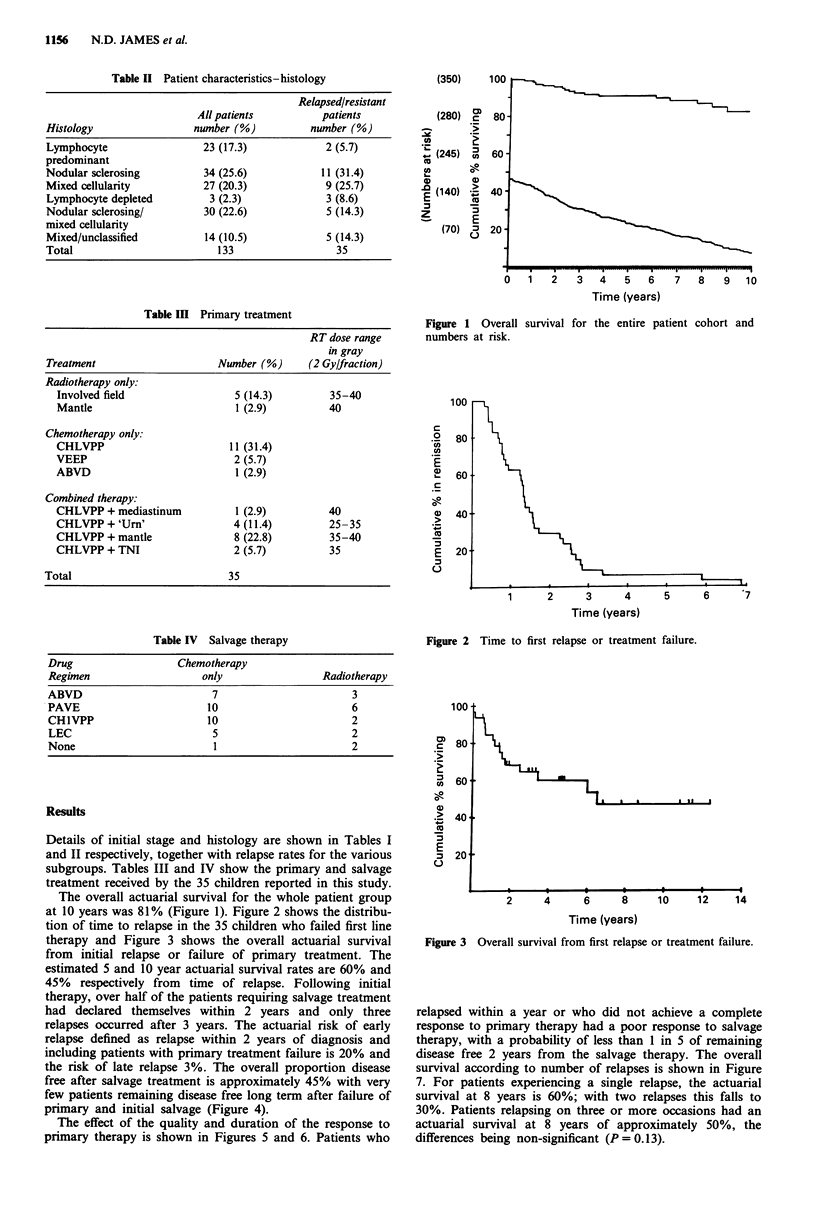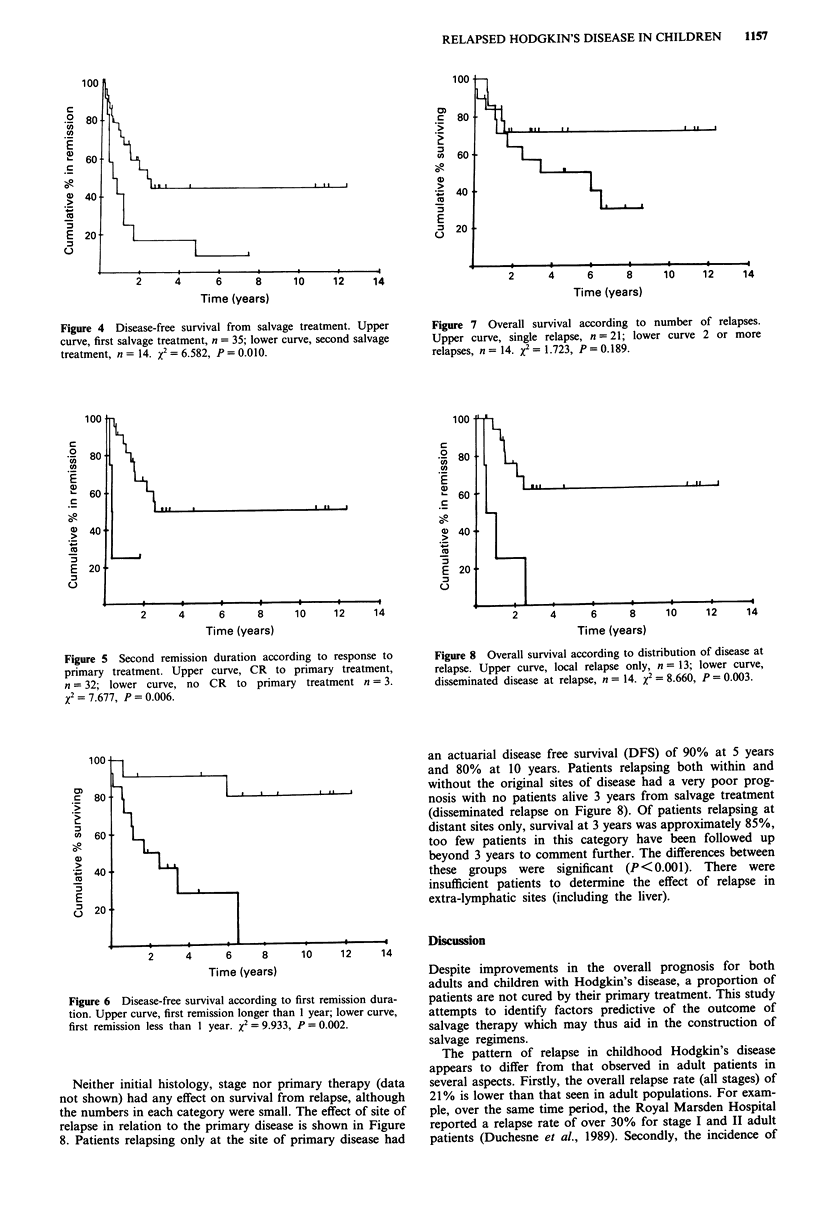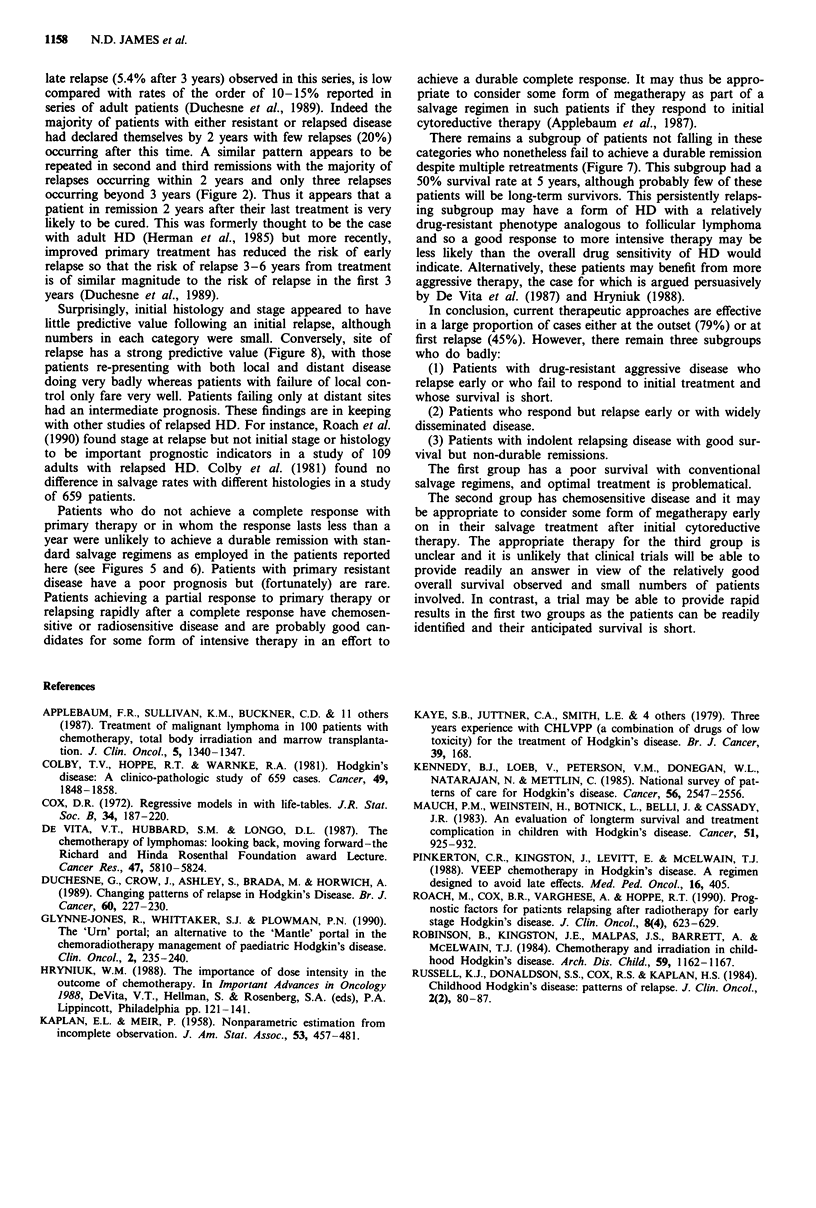# Outcome of children with resistant and relapsed Hodgkin's disease.

**DOI:** 10.1038/bjc.1992.426

**Published:** 1992-12

**Authors:** N. D. James, J. E. Kingston, P. N. Plowman, S. Meller, R. Pinkerton, A. Barrett, R. Sandland, T. J. McElwain, J. S. Malpas

**Affiliations:** Department of Paediatric Oncology, St Bartholomew's Hospital, West Smithfield, London, UK.

## Abstract

During the period 1974-89, 169 children with Hodgkin's disease were treated in the Paediatric Oncology Units of the Royal Marsden and St Bartholomew's Hospitals. The overall actuarial survival for the whole group was 81% at 10 years. Thirty-five of the 169 children either did not achieve a complete remission or subsequently relapsed. The estimated actuarial survival from initial relapse or failure of primary treatment was 60% at 5 years and 45% at 10 years. Over half of the patients requiring salvage therapy had declared themselves within 2 years and only 3 relapses occurred more than 3 years from diagnosis. Very few patients remain disease free long term after failure of primary and initial salvage therapy. Patients relapsing within a year of diagnosis or not achieving a complete response to primary therapy and those with disseminated relapse had a poor response to salvage therapy. A significant subgroup of patients had prolonged survival despite multiple relapses. Neither initial histology nor stage affected survival from relapse although numbers in each subgroup were small.


Br. J. Cancer (1992), 66, 1155–1158 © Macmillan Press Ltd., 1992

### Outcome of children with resistant and relapsed Hodgkin's disease

N.D. James[1]*, J.E. Kingston[1], P.N. Plowman[1], S. Meller[2], R. Pinkerton[2], A. Barrett[2], R. Sandland[2], T.J. McElwain[2] & J.S. Malpas[1]

[1]*Department of Paediatric Oncology, St Bartholomew's Hospital, West Smithfield, London EC1A 7BE, UK;* [2]*Department of Paediatric Oncology, Royal Marsden Hospital, Sutton, Surrey SM2 5PT, UK.*

**Summary** During the period 1974–89, 169 children with Hodgkin's disease were treated in the Paediatric Oncology Units of the Royal Marsden and St Bartholomew's Hospitals. The overall actuarial survival for the whole group was 81% at 10 years. Thirty-five of the 169 children either did not achieve a complete remission or subsequently relapsed. The estimated actuarial survival from initial relapse or failure of primary treatment was 60% at 5 years and 45% at 10 years. Over half of the patients requiring salvage therapy had declared themselves within 2 years and only 3 relapses occurred more than 3 years from diagnosis. Very few patients remain disease free long term after failure of primary and initial salvage therapy. Patients relapsing within a year of diagnosis or not achieving a complete response to primary therapy and those with disseminated relapse had a poor response to salvage therapy. A significant subgroup of patients had prolonged survival despite multiple relapses. Neither initial histology nor stage affected survival from relapse although numbers in each subgroup were small.

There are approximately 70 new cases of Hodgkin's Disease (HD) annually in the UK in children under the age of 15 years. The overall 5-year survival rate in this age group (all stages) is in excess of 80%. This figure falls with increasing age to less than 40% for those over 65 years at diagnosis (Kennedy *et al.*, 1985). Whilst factors predictive of relapse in both adults (Kennedy *et al.*, 1985) and children (Mauch *et al.*, 1983; Robinson *et al.*, 1984; Russell *et al.*, 1984); are well documented, there have been few studies looking at prognostic factors for those patients who relapse following primary therapy. We have undertaken a study of all children treated at the Royal Marsden and St Bartholomew's Hospitals during the period 1974–89, during which time common primary treatment programmes were in operation. In this paper we report our experience with relapsed or resistant HD and identify factors predictive of response to salvage treatment. Eighty-four of these patients have been previously analysed to assess factors predictive of a poor response to primary treatment (Robinson *et al.*, 1984).

### Methods

During the period 1974–89, a total of 169 patients aged less than 16 years were treated for HD. Of these patients, 35 either did not achieve a complete remission or subsequently relapsed and are the subject of this analysis. Treatment policies have changed over the study period as outlined below. Early stage cases (stage IA and some IIA) received primary radiotherapy either to involved sites only or to a full mantle to a dose of 35 Gy in 20 fractions sometimes with a boost to sites of bulk disease. During the period 1974–77, these early stage cases also received 3 courses of chemotherapy. Between 1974 and 1986, primary chemotherapy for HD comprised combination chemotherapy with chlorambucil, vinblastine, procarbazine, prednisolone (CHLVPP) (Kaye *et al.*, 1979). From 1987, in an effort to preserve fertility this regimen was replaced by vincristine, epirubicin, etoposide and prednisolone (VEEP) (Pinkerton *et al.*, 1988). The indications for radiotherapy as sole treatment remained unchanged. Between 1974 and 1990 patients with bulky mediastinal disease received adjuvant mediastinal radiotherapy to a dose of 25–35 Gy in 15–20 fractions (Glynne-Jones *et al.*, 1990). More recently, patients with IIA disease just involving upper cervical nodes have received radiotherapy alone.

A wide variety of salvage regimens were employed during the study period including prednisolone, doxorubicin, vincristine and etoposide (HOPE), HOPE + bleomycin (HOPE-BLEO), chlorambucil, etoposide and CCNU (LEC), high-dose melphalan with or without etoposide and BCNU followed by autologous bone marrow transplantation (ABMT), doxorubicin, bleomycin, vincristine and dacarbazine (ABVD), VEEP and CHLVPP with or without radiotherapy. The details of the stage, histology, primary and salvage treatments of the 35 patients at presentation are shown in Tables I–IV.

The effects of various parameters on the response to salvage treatment were determined using the log-rank method (Kaplan & Meir, 1958), multivariate analysis was by Cox regression (Cox, 1972). In order to simplify the analysis, remission duration was defined as the time from commencement of treatment to the date at which the treatment was deemed to have failed and thus includes patients who did not respond. In those cases in whom the disease clearly was not responsive this is presented as a very short 'remission'. This approach, whilst not strictly accurate, allows resistant and relapsed disease to be analysed together rather than in separate subgroups which would have resulted in very fragmented data. Where there are sufficient data, these two groups have been analysed separately.

**Table I** Patient characteristics

| *Stage* | *All patients number (%)* | *Relapsed/resistant patients number (%)* |
|---|---|---|
| 1A | 28 (21.1) | 4 (11.4) |
| 2A | 46 (34.6) | 12 (34.3) |
| 2B | 12 (9) | 3 (8.6) |
| 3A | 13 (9.8) | 5 (14.3) |
| 3AS | 8 (6.0) | 0 |
| 3B | 3 (2.3) | 1 (2.9) |
| 3BS | 5 (3.8) | 2 (5.7) |
| 4A | 3 (2.3) | 1 (2.9) |
| 4B | 15 (11.3) | 7 (20.0) |

Total number of patients = 169. Of these, 35 patients relapsed ($n = 32$) or had resistant disease ($n = 3$); 25 (71%) male, 10 (29%) female.

Correspondence: J.E. Kingston.
*Present address: Ludwig Institute for Cancer Research, St Mary's Hospital Medical School, Norfolk Place, London W2 1PG, UK.
Received 2 March 1992; and in revised form 3 June 1992.

Table II Patient characteristics–histology

| Histology | All patients number (%) | Relapsed/resistant patients number (%) |
|---|---|---|
| Lymphocyte predominant | 23 (17.3) | 2 (5.7) |
| Nodular sclerosing | 34 (25.6) | 11 (31.4) |
| Mixed cellularity | 27 (20.3) | 9 (25.7) |
| Lymphocyte depleted | 3 (2.3) | 3 (8.6) |
| Nodular sclerosing/ mixed cellularity | 30 (22.6) | 5 (14.3) |
| Mixed/unclassified | 14 (10.5) | 5 (14.3) |
| Total | 133 | 35 |

Table III Primary treatment

| Treatment | Number (%) | RT dose range in gray (2 Gy/fraction) |
|---|---|---|
| *Radiotherapy only:* | | |
| Involved field | 5 (14.3) | 35–40 |
| Mantle | 1 (2.9) | 40 |
| *Chemotherapy only:* | | |
| CHLVPP | 11 (31.4) | |
| VEEP | 2 (5.7) | |
| ABVD | 1 (2.9) | |
| *Combined therapy:* | | |
| CHLVPP + mediastinum | 1 (2.9) | 40 |
| CHLVPP + 'Urn' | 4 (11.4) | 25–35 |
| CHLVPP + mantle | 8 (22.8) | 35–40 |
| CHLVPP + TNI | 2 (5.7) | 35 |
| Total | 35 | |

Table IV Salvage therapy

| Drug Regimen | Chemotherapy only | Radiotherapy |
|---|---|---|
| ABVD | 7 | 3 |
| PAVE | 10 | 6 |
| CHIVPP | 10 | 2 |
| LEC | 5 | 2 |
| None | 1 | 2 |

### Results

Details of initial stage and histology are shown in Tables I and II respectively, together with relapse rates for the various subgroups. Tables III and IV show the primary and salvage treatment received by the 35 children reported in this study.

The overall actuarial survival for the whole patient group at 10 years was 81% (Figure 1). Figure 2 shows the distribution of time to relapse in the 35 children who failed first line therapy and Figure 3 shows the overall actuarial survival from initial relapse or failure of primary treatment. The estimated 5 and 10 year actuarial survival rates are 60% and 45% respectively from time of relapse. Following initial therapy, over half of the patients requiring salvage treatment had declared themselves within 2 years and only three relapses occurred after 3 years. The actuarial risk of early relapse defined as relapse within 2 years of diagnosis and including patients with primary treatment failure is 20% and the risk of late relapse 3%. The overall proportion disease free after salvage treatment is approximately 45% with very few patients remaining disease free long term after failure of primary and initial salvage (Figure 4).

The effect of the quality and duration of the response to primary therapy is shown in Figures 5 and 6. Patients who relapsed within a year or who did not achieve a complete response to primary therapy had a poor response to salvage therapy, with a probability of less than 1 in 5 of remaining disease free 2 years from the salvage therapy. The overall survival according to number of relapses is shown in Figure 7. For patients experiencing a single relapse, the actuarial survival at 8 years is 60%; with two relapses this falls to 30%. Patients relapsing on three or more occasions had an actuarial survival at 8 years of approximately 50%, the differences being non-significant ($P = 0.13$).

**Figure 1** Overall survival for the entire patient cohort and numbers at risk.

**Figure 2** Time to first relapse or treatment failure.

**Figure 3** Overall survival from first relapse or treatment failure.

**Figure 4** Disease-free survival from salvage treatment. Upper curve, first salvage treatment, $n = 35$; lower curve, second salvage treatment, $n = 14$. $\chi^2 = 6.582$, $P = 0.010$.

**Figure 5** Second remission duration according to response to primary treatment. Upper curve, CR to primary treatment, $n = 32$; lower curve, no CR to primary treatment $n = 3$. $\chi^2 = 7.677$, $P = 0.006$.

**Figure 6** Disease-free survival according to first remission duration. Upper curve, first remission longer than 1 year; lower curve, first remission less than 1 year. $\chi^2 = 9.933$, $P = 0.002$.

Neither initial histology, stage nor primary therapy (data not shown) had any effect on survival from relapse, although the numbers in each category were small. The effect of site of relapse in relation to the primary disease is shown in Figure 8. Patients relapsing only at the site of primary disease had

**Figure 7** Overall survival according to number of relapses. Upper curve, single relapse, $n = 21$; lower curve 2 or more relapses, $n = 14$. $\chi^2 = 1.723$, $P = 0.189$.

**Figure 8** Overall survival according to distribution of disease at relapse. Upper curve, local relapse only, $n = 13$; lower curve, disseminated disease at relapse, $n = 14$. $\chi^2 = 8.660$, $P = 0.003$.

an actuarial disease free survival (DFS) of 90% at 5 years and 80% at 10 years. Patients relapsing both within and without the original sites of disease had a very poor prognosis with no patients alive 3 years from salvage treatment (disseminated relapse on Figure 8). Of patients relapsing at distant sites only, survival at 3 years was approximately 85%, too few patients in this category have been followed up beyond 3 years to comment further. The differences between these groups were significant ($P < 0.001$). There were insufficient patients to determine the effect of relapse in extra-lymphatic sites (including the liver).

### Discussion

Despite improvements in the overall prognosis for both adults and children with Hodgkin's disease, a proportion of patients are not cured by their primary treatment. This study attempts to identify factors predictive of the outcome of salvage therapy which may thus aid in the construction of salvage regimens.

The pattern of relapse in childhood Hodgkin's disease appears to differ from that observed in adult patients in several aspects. Firstly, the overall relapse rate (all stages) of 21% is lower than that seen in adult populations. For example, over the same time period, the Royal Marsden Hospital reported a relapse rate of over 30% for stage I and II adult patients (Duchesne *et al.*, 1989). Secondly, the incidence of late relapse (5.4% after 3 years) observed in this series, is low compared with rates of the order of 10–15% reported in series of adult patients (Duchesne *et al.*, 1989). Indeed the majority of patients with either resistant or relapsed disease had declared themselves by 2 years with few relapses (20%) occurring after this time. A similar pattern appears to be repeated in second and third remissions with the majority of relapses occurring within 2 years and only three relapses occurring beyond 3 years (Figure 2). Thus it appears that a patient in remission 2 years after their last treatment is very likely to be cured. This was formerly thought to be the case with adult HD (Herman *et al.*, 1985) but more recently, improved primary treatment has reduced the risk of early relapse so that the risk of relapse 3–6 years from treatment is of similar magnitude to the risk of relapse in the first 3 years (Duchesne *et al.*, 1989).

Surprisingly, initial histology and stage appeared to have little predictive value following an initial relapse, although numbers in each category were small. Conversely, site of relapse has a strong predictive value (Figure 8), with those patients re-presenting with both local and distant disease doing very badly whereas patients with failure of local control only fare very well. Patients failing only at distant sites had an intermediate prognosis. These findings are in keeping with other studies of relapsed HD. For instance, Roach *et al.* (1990) found stage at relapse but not initial stage or histology to be important prognostic indicators in a study of 109 adults with relapsed HD. Colby *et al.* (1981) found no difference in salvage rates with different histologies in a study of 659 patients.

Patients who do not achieve a complete response with primary therapy or in whom the response lasts less than a year were unlikely to achieve a durable remission with standard salvage regimens as employed in the patients reported here (see Figures 5 and 6). Patients with primary resistant disease have a poor prognosis but (fortunately) are rare. Patients achieving a partial response to primary therapy or relapsing rapidly after a complete response have chemosensitive or radiosensitive disease and are probably good candidates for some form of intensive therapy in an effort to achieve a durable complete response. It may thus be appropriate to consider some form of megatherapy as part of a salvage regimen in such patients if they respond to initial cytoreductive therapy (Applebaum *et al.*, 1987).

There remains a subgroup of patients not falling in these categories who nonetheless fail to achieve a durable remission despite multiple retreatments (Figure 7). This subgroup had a 50% survival rate at 5 years, although probably few of these patients will be long-term survivors. This persistently relapsing subgroup may have a form of HD with a relatively drug-resistant phenotype analogous to follicular lymphoma and so a good response to more intensive therapy may be less likely than the overall drug sensitivity of HD would indicate. Alternatively, these patients may benefit from more aggressive therapy, the case for which is argued persuasively by De Vita *et al.* (1987) and Hryniuk (1988).

In conclusion, current therapeutic approaches are effective in a large proportion of cases either at the outset (79%) or at first relapse (45%). However, there remain three subgroups who do badly:

(1) Patients with drug-resistant aggressive disease who relapse early or who fail to respond to initial treatment and whose survival is short.

(2) Patients who respond but relapse early or with widely disseminated disease.

(3) Patients with indolent relapsing disease with good survival but non-durable remissions.

The first group has a poor survival with conventional salvage regimens, and optimal treatment is problematical.

The second group has chemosensitive disease and it may be appropriate to consider some form of megatherapy early on in their salvage treatment after initial cytoreductive therapy. The appropriate therapy for the third group is unclear and it is unlikely that clinical trials will be able to provide readily an answer in view of the relatively good overall survival observed and small numbers of patients involved. In contrast, a trial may be able to provide rapid results in the first two groups as the patients can be readily identified and their anticipated survival is short.

### References

APPLEBAUM, F.R., SULLIVAN, K.M., BUCKNER, C.D. & 11 others (1987). Treatment of malignant lymphoma in 100 patients with chemotherapy, total body irradiation and marrow transplantation. *J. Clin. Oncol.*, **5**, 1340–1347.

COLBY, T.V., HOPPE, R.T. & WARNKE, R.A. (1981). Hodgkin's disease: A clinico-pathologic study of 659 cases. *Cancer*, **49**, 1848–1858.

COX, D.R. (1972). Regressive models in with life-tables. *J.R. Stat. Soc. B*, **34**, 187–220.

DE VITA, V.T., HUBBARD, S.M. & LONGO, D.L. (1987). The chemotherapy of lymphomas: looking back, moving forward–the Richard and Hinda Rosenthal Foundation award Lecture. *Cancer Res.*, **47**, 5810–5824.

DUCHESNE, G., CROW, J., ASHLEY, S., BRADA, M. & HORWICH, A. (1989). Changing patterns of relapse in Hodgkin's Disease. *Br. J. Cancer*, **60**, 227–230.

GLYNNE-JONES, R., WHITTAKER, S.J. & PLOWMAN, P.N. (1990). The 'Urn' portal; an alternative to the 'Mantle' portal in the chemoradiotherapy management of paediatric Hodgkin's disease. *Clin. Oncol.*, **2**, 235–240.

HRYNIUK, W.M. (1988). The importance of dose intensity in the outcome of chemotherapy. In *Important Advances in Oncology 1988*, DeVita, V.T., Hellman, S. & Rosenberg, S.A. (eds), P.A. Lippincott, Philadelphia pp. 121–141.

KAPLAN, E.L. & MEIR, P. (1958). Nonparametric estimation from incomplete observation. *J. Am. Stat. Assoc.*, **53**, 457–481.

KAYE, S.B., JUTTNER, C.A., SMITH, L.E. & 4 others (1979). Three years experience with CHLVPP (a combination of drugs of low toxicity) for the treatment of Hodgkin's disease. *Br. J. Cancer*, **39**, 168.

KENNEDY, B.J., LOEB, V., PETERSON, V.M., DONEGAN, W.L., NATARAJAN, N. & METTLIN, C. (1985). National survey of patterns of care for Hodgkin's disease. *Cancer*, **56**, 2547–2556.

MAUCH, P.M., WEINSTEIN, H., BOTNICK, L., BELLI, J. & CASSADY, J.R. (1983). An evaluation of longterm survival and treatment complication in children with Hodgkin's disease. *Cancer*, **51**, 925–932.

PINKERTON, C.R., KINGSTON, J., LEVITT, E. & MCELWAIN, T.J. (1988). VEEP chemotherapy in Hodgkin's disease. A regimen designed to avoid late effects. *Med. Ped. Oncol.*, **16**, 405.

ROACH, M., COX, B.R., VARGHESE, A. & HOPPE, R.T. (1990). Prognostic factors for patients relapsing after radiotherapy for early stage Hodgkin's disease. *J. Clin. Oncol.*, **8(4)**, 623–629.

ROBINSON, B., KINGSTON, J.E., MALPAS, J.S., BARRETT, A. & MCELWAIN, T.J. (1984). Chemotherapy and irradiation in childhood Hodgkin's disease. *Arch. Dis. Child.*, **59**, 1162–1167.

RUSSELL, K.J., DONALDSON, S.S., COX, R.S. & KAPLAN, H.S. (1984). Childhood Hodgkin's disease: patterns of relapse. *J. Clin. Oncol.*, **2(2)**, 80–87.